# An intracranial yolk sac tumor with initial symptom of hemorrhage in the basal ganglia: a case report

**DOI:** 10.3389/fonc.2024.1402819

**Published:** 2024-12-06

**Authors:** Qiang Liu, Xuecui Du, Yunyan Wang, Ruihua Hou, Yuqing Chen, Teng Chen

**Affiliations:** ^1^ Department of Neurosurgery, Qilu Hospital of Shandong University, JiNan, China; ^2^ Department of Oncology, The Third People’s Hospital of Tai’an City, TaiAn, China; ^3^ Department of Psychiatry, Clinical and Experimental Sciences Faculty of Medicine University of Southampton, Southampton, United Kingdom; ^4^ School of Clinical Medicine, Addenbrooke’s Hospital, University of Cambridge, London, United Kingdom

**Keywords:** yolk sac tumor, intracranial germ-cell tumor, Wallerian degeneration, hemorrhage, basal ganglia

## Abstract

We report a case and follow-up of an adult male with intracranial yolk sac tumor (YST). Initially, the patient presented with abnormal high signals in the right basal ganglia on MRI, misdiagnosed as a cavernous hemangioma. However, within 2 years, the condition rapidly progressed into a large, hypervascular solid neoplasm leading to a basal ganglia hemorrhage. Comprehensive evaluation of clinical symptoms, imaging, surgical findings, serology, histopathology, and genetic analysis confirmed the diagnosis of a yolk sac tumor. The patient underwent prompt surgical resection followed by radiotherapy and chemotherapy. Six months post-treatment, his condition remains stable, with no recurrence. Notably, early MRI revealed Wallerian degeneration in the brainstem suggesting that benign-appearing basal ganglia lesions may sometimes result from malignant infiltration by germ cell tumors or other cancers. To prevent misdiagnosis and ensure timely treatment, a stereotactic biopsy is recommended. We hope this case provides a valuable reference for diagnosing and treating YSTs and contributes to ongoing research aimed at improving patient survival.

## Introduction

Yolk sac tumors (YSTs) typically originate in the gonads (ovary and testis), with extragonadal primary sites being extremely rare accounting for only 10%–20% of cases (1). YSTs primarily occur in children and adolescents, but are less common in adults (2). According to the WHO classification, primary YSTs in the brain is categorized as an intracranial germ cell tumor representing approximately 7% of all cases (3). The pineal gland and suprasellar region are the most common parts of intracranial germ cell tumors (GCTs). Recent reports have documented intracranial YSTs in the ventricle system, basal ganglia, and other parts of the cerebral and cerebellar hemispheres (4). However, primary YSTs in the basal ganglia have rarely been documented. Here, we present a case of primary intracranial YST with tumor-related hemorrhage detailing the patient’s clinical symptoms, diagnostic evaluation, differential diagnosis, surgical treatment, and follow-up prognosis.

## Case presentation

### Admission condition

The patient, a 22-year-old male, was admitted to the neurology ward on 8 March 2023 following a 10-day history of headache and vomiting, along with left upper limb weakness for 6 days. On examination, his axillary temperature was 38.2°C, with stable vital signs. He had a 5 × 5-cm stage 1 pressure ulcer in the sacral region and a 3 × 3-cm stage 1 ulcer on the left lateral malleolus, along with multiple abrasions on the left calf. His pharynx showed mild congestion, and lung auscultation was clear without abnormal sounds. The heart and abdomen revealed no significant abnormalities. Notably, his pupils were asymmetrically dilated (5 mm on the left, 3 mm on the right), though direct and indirect light reflexes were normal. He exhibited partial facial paralysis on the left side, with uncoordinated muscle strength and increased muscle tone in his left limb. Stiff neck, Kernig, and Brudzinski signs were positive, as were pathological reflexes. He also had urinary incontinence and constipation for over a week. The patient had a prior history of basal ganglia hemorrhage 2 years earlier initially diagnosed as a “cavernous hemangioma.” He was also a heavy smoker and drinker.

### Imaging examination

**Figure 1:**
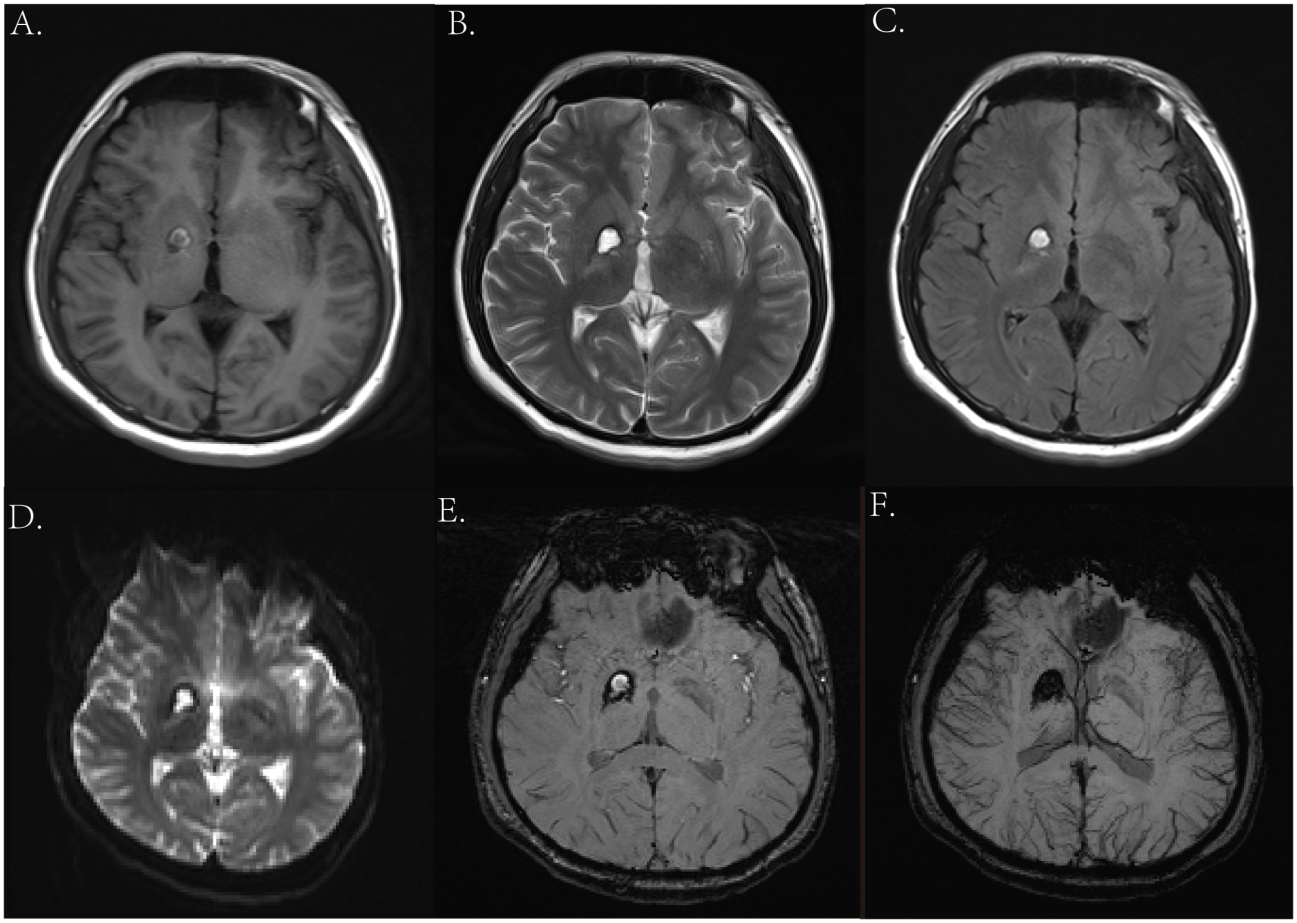
Magnetic resonance imaging (MRI) from previous admission 2 years ago. **(A)** Axial T1-weighted image. **(B)** Axial T2-weighted image. **(C)** Axial T2-FLAIR image. **(D)** Axial DWI image. **(E, F)** Axial SWI image.

**Figure 2:**
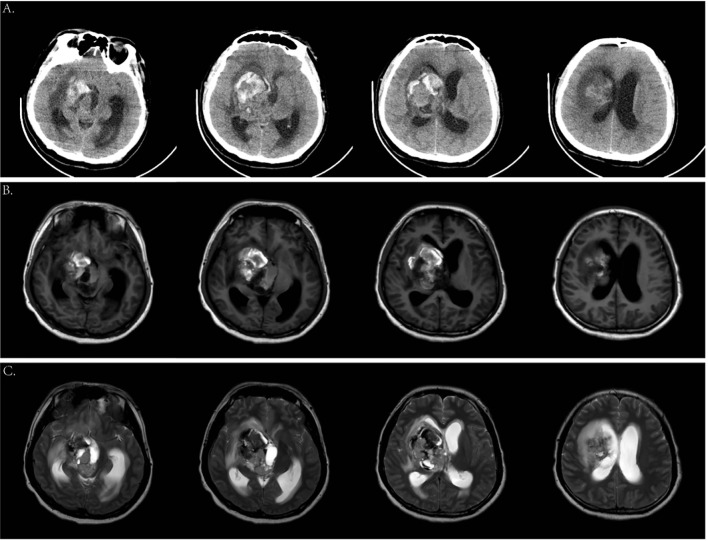
Cranial computed tomography (CT) scan and magnetic resonance imaging (MRI) on the current admission. **(A)** Emergency head CT scan showed an amount of hemorrhage in the right basal ganglia. **(B)** Axial T1-weighted images. **(C)** Axial T2-weighted images.

**Figure 3:**
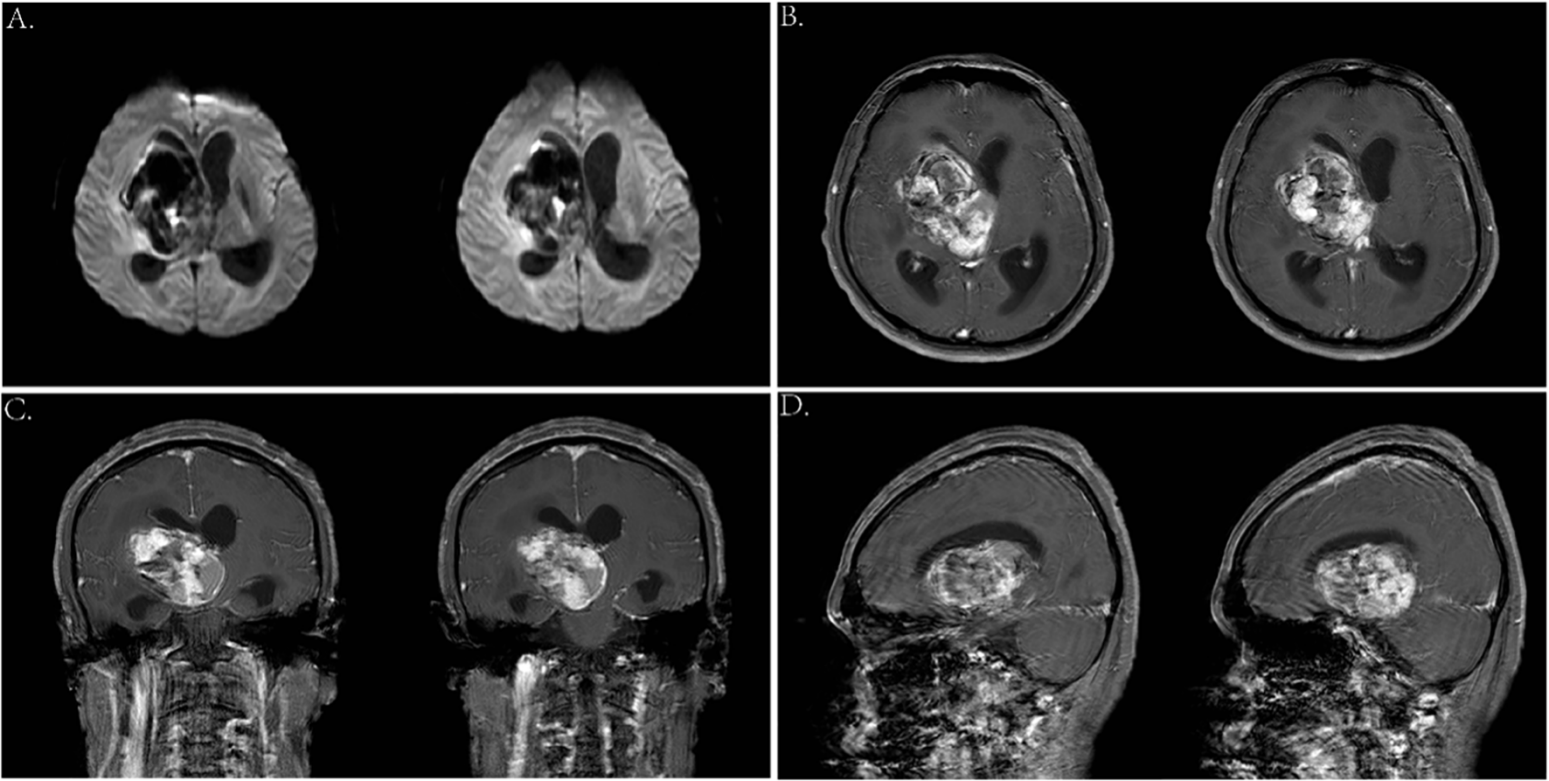
**(A)** Axial DWI images. **(B—D)** Enhanced MRI scan showed a 6.4 * 4.8 * 5.1-cm mass in the right basal ganglia.

Two years ago, the patient underwent brain MRI, including an SWI sequence, which revealed patchy lesions in the right basal ganglia measuring approximately 1.4 × 1.1 × 1.6 cm. T1-weighted imaging, T2-weighted imaging, T2-FLAIR, and DWI showed low peripheral and high internal signals (**Figures 1A–D**), with similar findings on SWI (**Figures 1E, F**). He was diagnosed with cavernous angioma during the recovery period from hemorrhage. However, after his current admission, an urgent brain CT revealed hemorrhage in the right basal ganglia and corona radiata, along with supratentorial ventricular enlargement. Neurology consultation initially suggested a ruptured arteriovenous malformation in the basal ganglia (**Figure 2A**), but a tumor was also considered. A follow-up enhanced brain MRI showed an irregular mass with heterogeneous signals: high on T1-weighted (**Figure 2B**), low signal in T2-wieghted (**Figure 2C**), and mixed on diffusion-weighted imaging (**Figure 3A**). The tumor measured 6.4 × 4.8 × 5.1 cm at its largest section (**Figures 3B–D**), with surrounding edema and protrusion into the midbrain aqueduct causing supratentorial ventricular dilation. Based on the CT and MRI findings, a hemorrhagic, hypervascular tumor in the right basal ganglia–corona radiata region was diagnosed. To rule out distant metastasis, a thoracic–abdominal–pelvic CT scan was performed revealing no abnormalities.

### Operation

Before the surgery, we assessed the patient’s heart, lung, and kidney functions, as well as his tolerance to anesthesia. The family was informed of the risks and potential complications. Following a multidisciplinary discussion, and at the strong request of his parents, we proceeded with “resection of the basal ganglia tumor via right frontotemporal craniotomy, evacuation of intracerebral hematoma, and lateral ventricular puncture and drainage” under general anesthesia on 16 March 2023.

**Figure 4:**
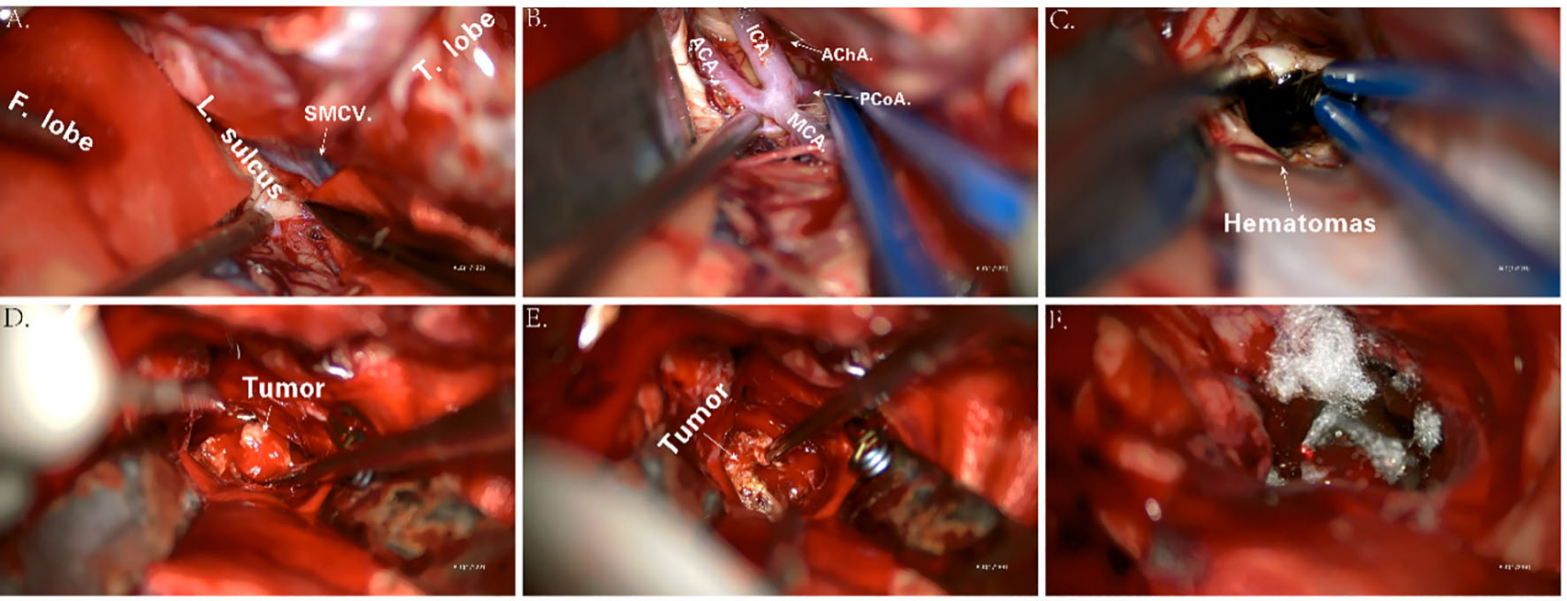
Key anatomic presentations during surgery **(A–F)**. F. lobe, frontal lobe; L. sulcus, lateral sulcus; SMCV, superficial middle cerebral vein; T. lobe, temporal lobe; ICA, internal carotid artery; ACA, anterior cerebral artery; MCA, middle cerebral artery; AChA, anterior choroidal artery; PCoA, posterior communicating artery.

The procedure began with marking the left para-midline followed by a 3-cm incision. A burr hole was drilled, and the ventricular end of the drainage tube was placed at the midpoint of the line between the bilateral external auditory canals. Yellowish cerebrospinal fluid with high pressure was noted, and the drainage tube was connected and clamped. Next, an arc incision was made in the right frontotemporal region exposing the frontal and temporal skulls. The dura was incised, and cerebrospinal fluid was released. As cerebral pressure decreased, the A1 segment of the anterior cerebral artery, middle cerebral artery, and its branches were fully exposed (**Figures 4A, B**). After protecting the surrounding arteries, yellow-stained insular cortex tissue was noted, and brown fluid under high pressure was aspirated, completely removing the hematoma (**Figure 4C**). Further exploration revealed a tough, highly vascular lesion invading the ipsilateral ventricular wall and surrounding basal ganglia. The lesion’s unclear boundary with normal brain tissue suggested a malignant tumor. Tumor decompression was achieved by resecting the tissue in blocks (**Figures 4D, E**). Portions of the tumor invading the ventricular wall were also removed, though the section closely related to the caudate nucleus could not be fully excised. Following complete hemostasis, a filament was placed in the cavity (**Figure 4F**), and a drainage tube was inserted. The dura was sutured, with the defect repaired using artificial material. The bone flap was secured with absorbable plates, a second drainage tube was placed under the skin, and the scalp was sutured and dressed. The patient received 4 U of red blood cells and 380 ml of plasma before being transferred to the ICU with endotracheal intubation.

### Tumor markers

**Figure 5:**
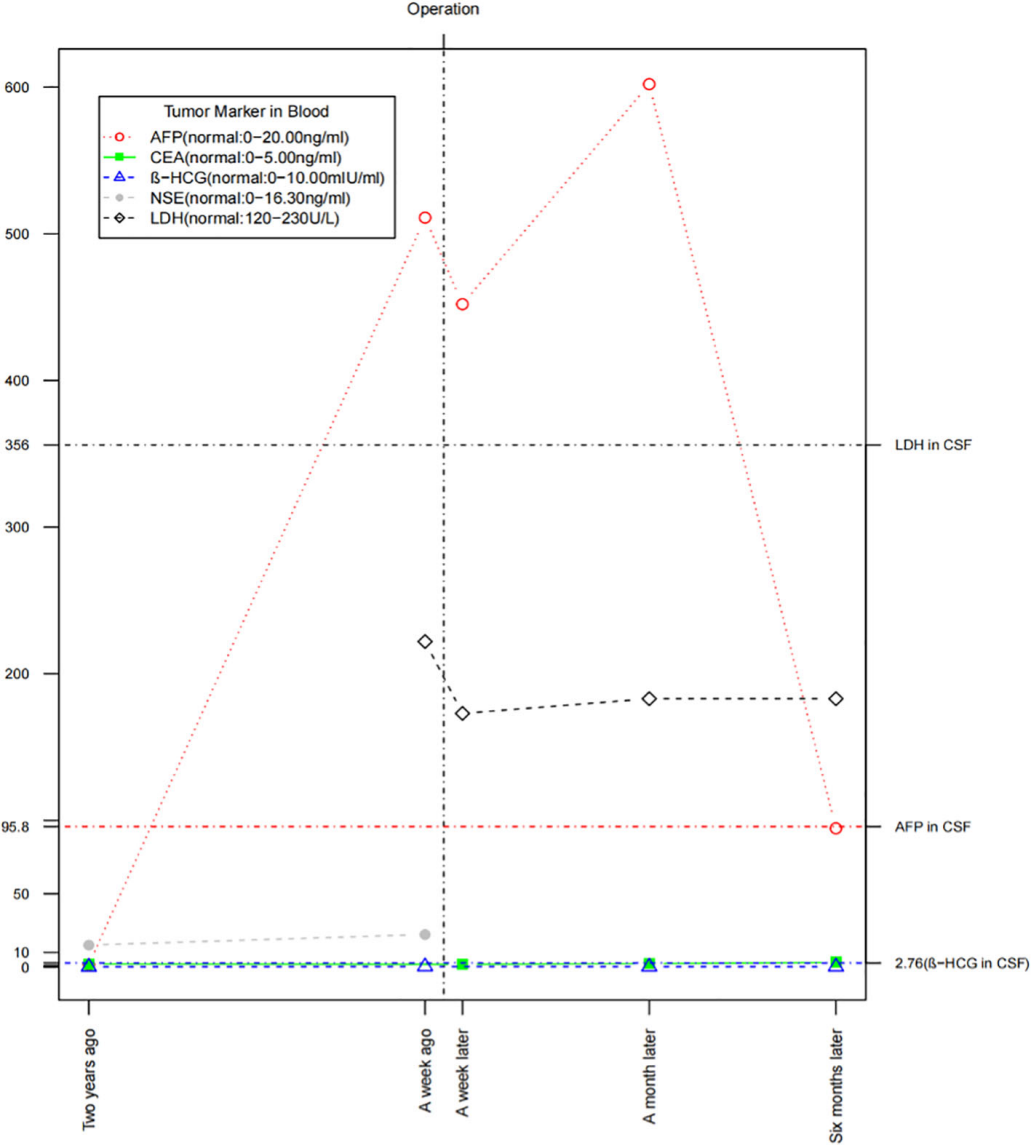
The fluctuation of tumor markers in the blood and cerebrospinal fluid.

We monitored the patient’s tumor markers (5) 2 years ago, a week before surgery, and at 1 week, 1 month, and 6 months post-surgery (**Figure 5**). The AFP level was closely correlated with the tumor’s progression, while cerebrospinal fluid LDH showed a slight increase. Serum β-HCG, CEA, and NSE remained within normal ranges. Notably, the patient had significantly low levels of FSH (0.69 mIU/ml, normal: 1.50–12.40 mIU/ml) and testosterone (0.93 nmol/L, normal: 6.68–29.00 nmol/L) suggesting the possibility of a germ cell tumor, such as a pineal tumor.

### Pathological examination

**Figure 6:**
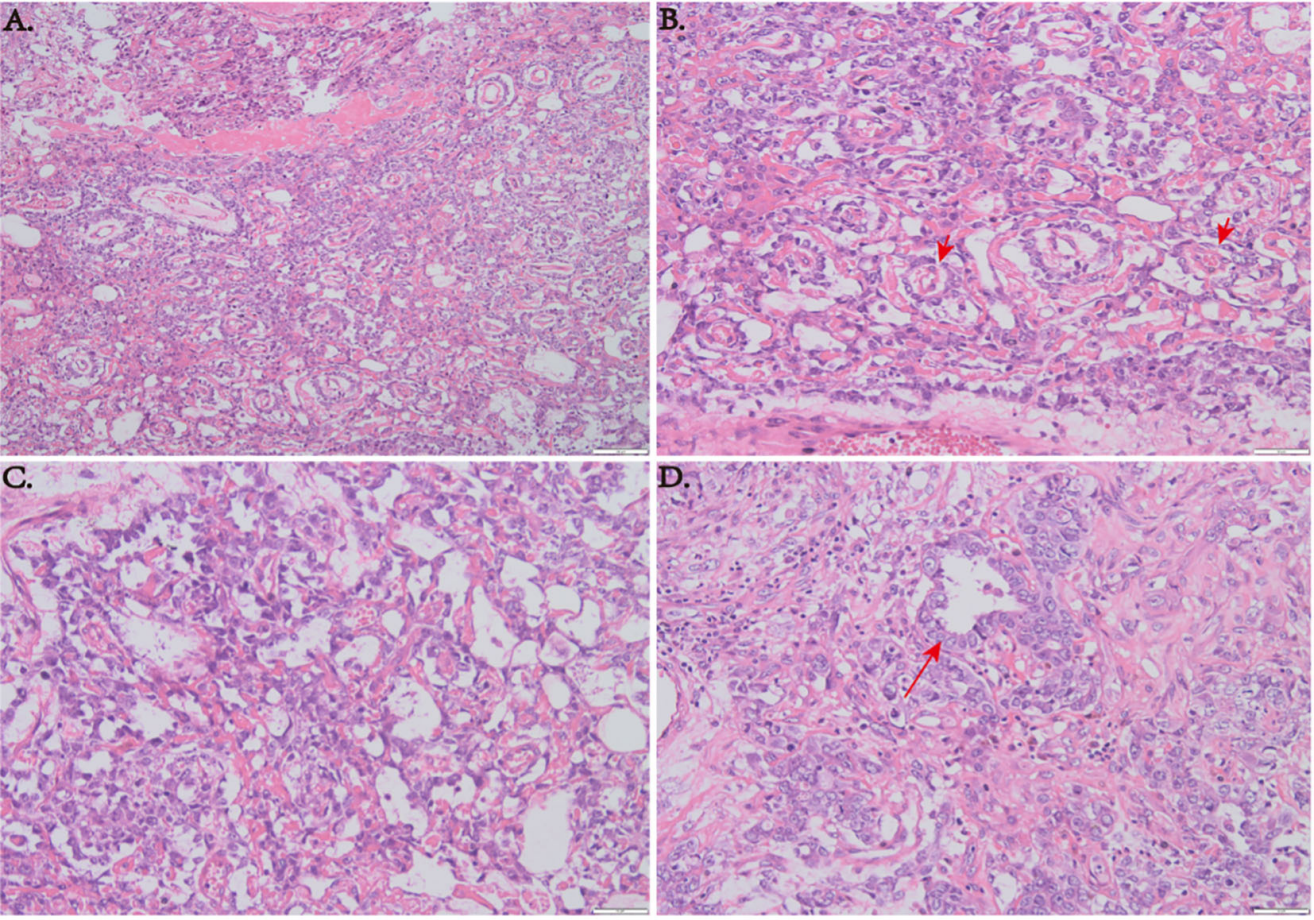
**(A)** HE staining at low magnification (×100) showing a sinusoid lacunar or reticular growth pattern of tumor cells. **(B)** Typical Schiller–Duval bodies. **(C)** Loose reticular structures (×200). **(D)** Acinar–adenoid structures (×200).

**Figure 7:**
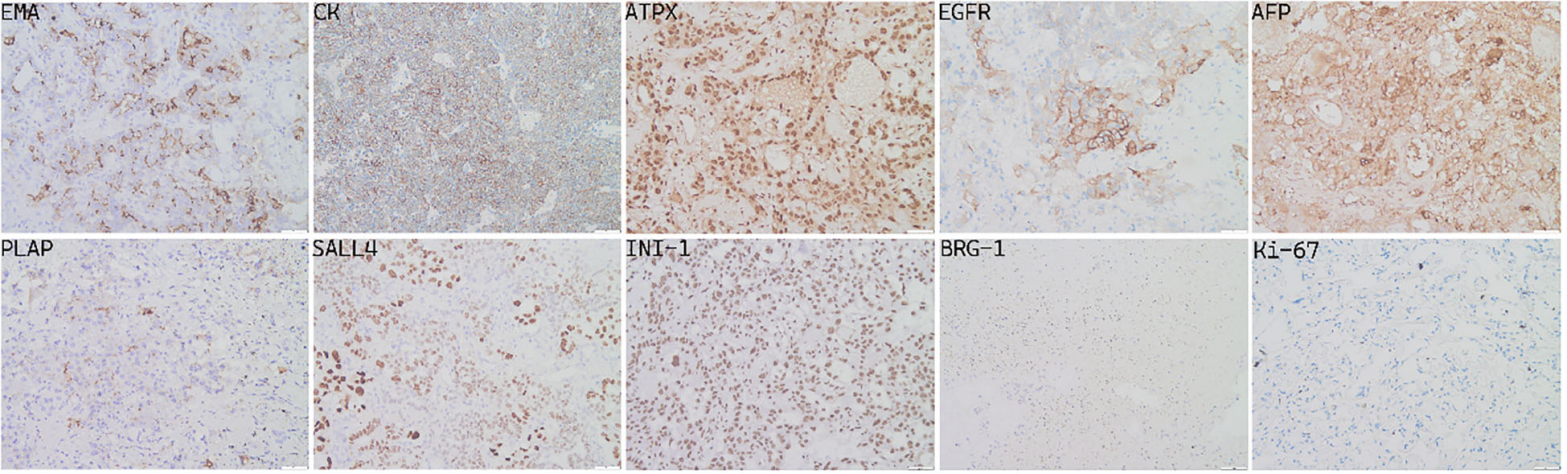
Positive immunohistochemical staining (×200).

The specimens removed during surgery consisted of gray or gray red tissue measuring approximately 5.5 cm × 1.2 cm, with a tough texture and surface clotting. HE staining (**Figure 6**) revealed clear, bright cells with pathological nuclear division under the microscope, arranged in sinusoidal, lacunar, and reticular patterns. Typical Schiller–Duval bodies, loose reticular structures, and acinar–adenoid formations were observed easily. Immunohistochemistry showed the following (**Figure 7**): EMA (local +), CK (+), GFAP (−), OLig-2 (−), NeuN (−), ATRX (+), Syn (−), EGFR (+), OCT-4 (−), CD30 (−), AFP (+), PLAP (local +), SALL 4 (+), CD117 (−), INI-1 (+), BRG-1 (+), Ki-67 (+, 50%) (6–8). Based on these findings, the final diagnosis was yolk sac tumor.

### Molecular genetic examination

**Figure 8:**
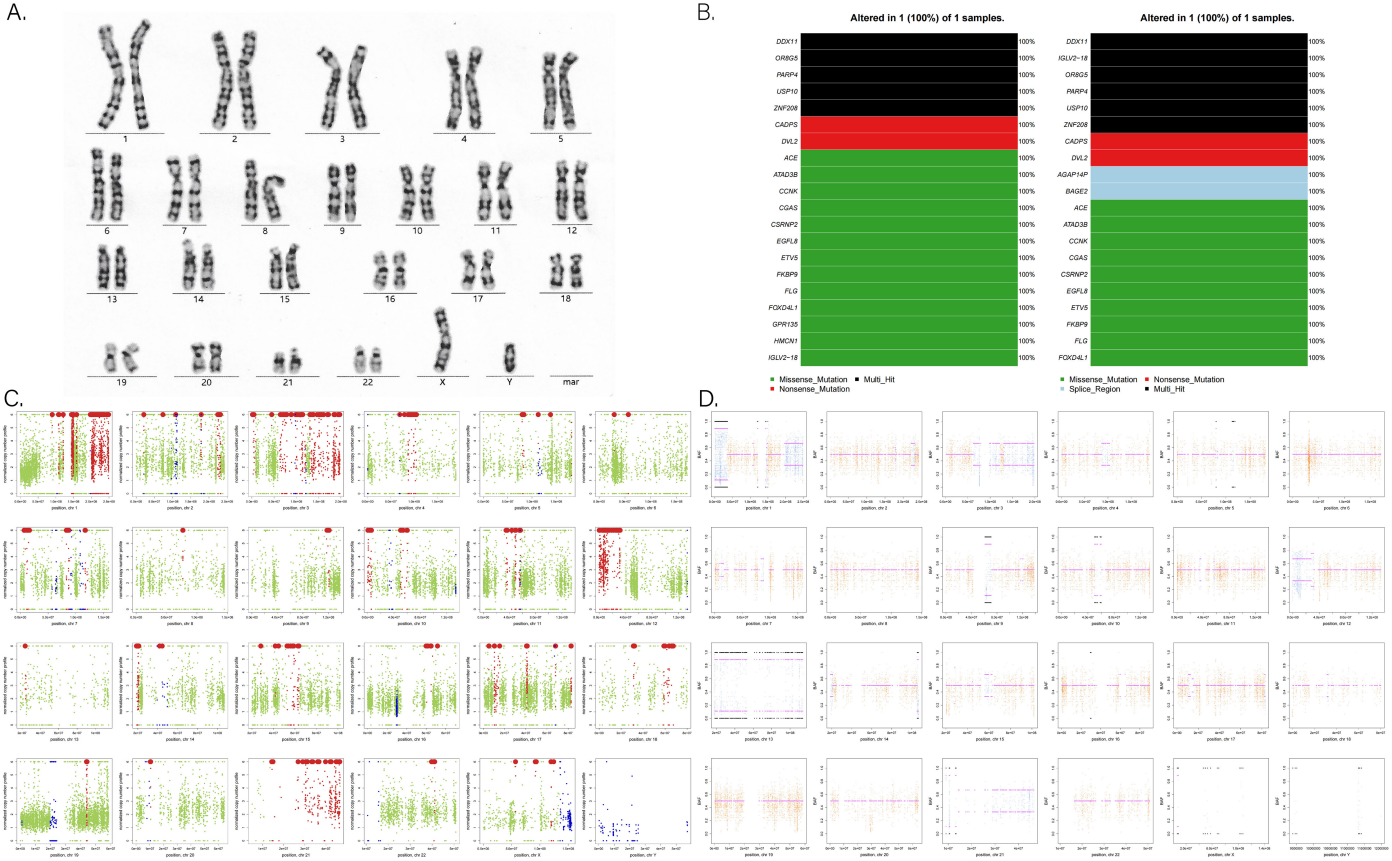
**(A)** G-banded chromosome karyotype. **(B)** Tumor-driver genes based on OncodriveCLUST (left) and OncodriveFM (right) prediction models. **(C)** Analysis of somatic CNV (copy number variation) in chromosomes. Red points mean an increase in copy number; blue points mean a reduction in copy number; other data are marked in green points. **(D)** Analysis of somatic LOH (loss of heterozygosity) in chromosomes. Orange indicates a uniform AB allele distribution, and blue indicates a preference for AB allele distribution, where loss of heterozygosity occurred.

Previous studies have reported an increased incidence of YSTs in men with Klinefelter syndrome (47, XXY) (9) and in individuals with trisomy 21 (Down syndrome) (10). Molecular biological studies have also revealed abnormal genome copies and loss of heterozygosity in intracranial GCTs (11). Given the patient**’**s family history of spontaneous abortion and infant death, we investigated potential genetic abnormalities. After consulting with his family, blood and tumor samples were collected for genomic exon sequencing. Although the karyotype was normal, significant somatic genomic instability was observed, including copy number variation (CNV) and loss of heterozygosity (LOH) (**Figures 8A, C, D**). Genomic data were further analyzed using OncodriveCLUST (12) and OncodriveFM (13) scoring and comparing each gene based on these models. The top 20 driver genes promoting tumor progression and their associated mutation types were predicted (**Figure 8B**).

### Treatment and follow-up

**Figure 9:**
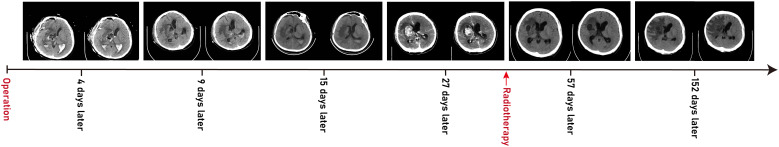
The change in head CT after operation.

Postoperatively, external ventricular drainage was used to clear operative area bleeding, and mannitol was administered to reduce intracranial pressure, along with sedation, antibiotics, and nutritional support. The dressing was changed every 2–3 days, and the ventricular drainage tube was removed after 1 week. However, multiple CT scans revealed a lateral ventricle hematoma with mild dilation (**Figure 9**) prompting the decision to insert a lumbar puncture tube for continuous external lumbar drainage. After 1 week of cerebrospinal fluid drainage, the patient’s consciousness gradually improved, though residual left limb weakness persisted (muscle strength grade 3). In consultation with the oncologist, radiotherapy and chemotherapy were recommended to reduce the risk of tumor recurrence. The patient was discharged on postoperative day 18. During the 6-month follow-up, the patient initially received BEP chemotherapy (bleomycin–etoposide–cisplatin) at the local hospital, but treatment was discontinued due to severe side effects and replaced with whole-brain radiotherapy (40–50 Gy). To date, he has completed 30 sessions of radiotherapy and is in generally good condition, able to get out of bed, but still experiences mild confusion and cognitive impairment. We will continue long-term follow-up to monitor his prognosis.

## Discussion

In 1959, *G. Teilum* (14) found a tumor in a testis and an ovary with a tissue structure similar to the endodermal sinus of rat placenta naming it “Endodermal sinus tumor” for the first time. Subsequent clinical case reports and histopathological evidence led to the conclusion that these tumors originate from residual yolk sac tissue from the third to fourth week of embryonic development. Under certain conditions, these cells can continuously grow and differentiate (15, 16). Typically, the yolk sac migrates and degenerates to the gonadal crest during embryonic development contributing to the formation of primordial germ cells, which is why 80% of YST occur in the gonads. However, during migration, some primordial germ cells may remain in or migrate to body cavity tissues or ectodermal trophoblast tissues leading to extragonadal YST in locations, like the abdominal cavity, thoracic cavity, or craniocerebral region, accounting for approximately 10%–15% of cases (17). Additionally, dislocation of pluripotent embryonic cells into the lateral mesoderm or abnormal aggregation in different brain regions may contribute to the occurrence of intracranial YSTs (18). For instance, Lee et al. have found that endogenous neural stem cells in the brain can be induced to differentiate into YST (19).

The clinical manifestations of intracranial YSTs often lack specificity. Similar to other brain tumors, the symptoms are closely related to the size and location of the mass primarily manifesting as compression of adjacent tissues and space-occupying effect. Tumors originating in the pineal region, like pinealoma, can compress or obstruct the cerebral aqueduct leading to increased intracranial pressure, drowsiness, abnormal visual field, epilepsy, and ataxia (20). Approximately 75% of these tumors invade the quadrigeminal plate causing Parinaud syndrome characterized by binocular vision disturbances and convergence dysfunction. Tumors in the sellar region, like pituitary adenoma or craniopharyngioma, often affect the optic chiasm and nerves resulting in vision impairment, loss, or even blindness. They can also damage the hypothalamic–pituitary axis causing precocious puberty, sexual retardation, diabetes insipidus, and pituitary failure (21). Tumors located in the basal ganglia and lateral ventricles, such as ependymoma or glioma, are associated with hemiplegia, fever of unknown origin, visual symptoms, and obstructive hydrocephalus (mental symptoms, convulsions, precocious puberty, diabetes insipidus, etc.). Moreover, the duration of symptoms vary from 1 month to 4.5 years, with an average of 1.5 years (22–25). However, this case was unique, as hemorrhage in the basal ganglia was the first symptom, a presentation rarely mentioned in the existing reports of YST (10, 26–29). This increased the difficulty of differential diagnosis, and the final diagnosis was confirmed via craniotomy.

**Figure 10:**
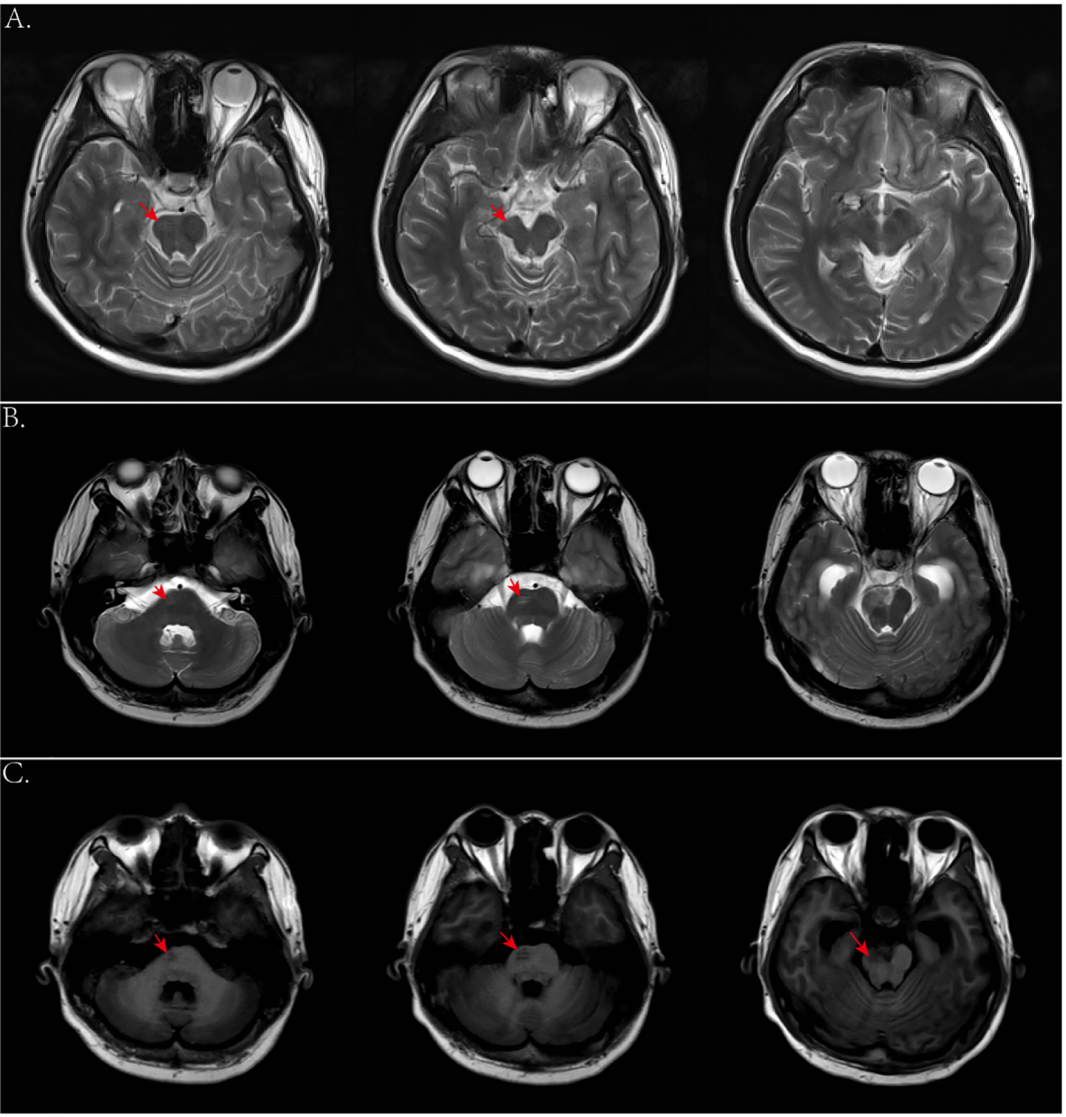
**(A)** Axial T2-weighted images 2 years ago showed mild atrophy of the brainstem on the affected side. **(B, C)** Axial T2- and T1-weighted images this time showed degeneration and damage of the brainstem conduction fiber bundles on the affected side.

Due to the rarity of yolk sac tumor, characteristic clinical imaging features have not been well established. For example, tumors with a rich blood supply are often associated with hemorrhage leading to high or mixed density and irregular shapes on initial head CT scans. In this case, the hemorrhage was misinterpreted as being caused by a ruptured vascular malformation. In addition, the hemorrhage manifestations could also mask the symptoms of the tumor. However, enhanced MRI can differentiate between tumors and vascular malformations, such as AVMs and cavernous hemangiomas. When a mass is not yet formed in the early stages, PET-CT or stereotactic biopsy is recommended if conditions permitted. In addition, we observed significant Wallerian degeneration on the patient’s MRI, presenting as T1 or long T1 and long T2 signals along the pyramidal tract strip on the side of the lesion (**Figures 10B, C**), and mild brainstem atrophy on the affected side (22–24, 30) (**Figure 10A**). That suggested pyramidal tract damage caused by tumor infiltration into the internal capsule or thalamus (31, 32). In some cases, atrophy of the ipsilateral cerebral hemisphere has also been observed (33). This typical MRI manifestation might be a characteristic feature of germinomas in the basal ganglia region.

According to reported clinicopathological data, YSTs are composed of primitive epithelial cells resembling yolk sac endoderm. Their hallmarked morphological feature is Schiller–Duval body, which is similar to glomerular structures composed of monolayer cuboidal or columnar tumor cells and formed around capillaries, thin-walled sinuses, or venules (17). Meanwhile, the common histological features include loose reticular or sinusoidal-like structures, acinar–glandular tube-like formations, eosinophilic hyaline bodies, and solid nest-like structures composed of basement membrane-like material. Generally, the immunohistochemical expression of AFP, CK, PLAP, SALL4, C-kit, Glypican 3 (membrane heparan sulfate glycoprotein) was positive, CD117 was partly positive, but OCT3/4, CD30, and β-HCG were negative. Carcinoembryonic antigen (CEA), CK-Pan, CK7, and EMA were expressed positively in tumor cells with adenoid differentiation (34), as confirmed in this case (**Figure 7**). Other studies had shown that HNF1β was significantly expressed in yolk sac tumor with a sensitivity of 100% and a specificity of 80% (35). Xiao et al. also proposed a new sensitive marker ZBTB16, with a sensitivity of 100% and a specificity of 66% for diagnosing of YSTs (36). Almost all YST patients exhibit elevated AFP levels in serum and cerebrospinal fluid, likely due to tumor cells retaining the ability to synthesize AFP, as seen in embryonic yolk sac. Talerman et al. demonstrated that AFP is a specific marker for YSTs playing a crucial role in its preoperative diagnosis and prognosis (37).

Despite few researches on the molecular pathogenesis of intracranial GCTs, early studies have highlighted chromosomal instability, particularly involving isochromosome 12p. Fluorescence *in situ* hybridization has demonstrated amplification at 12p13 in nearly all intracranial GCTs, regardless of histological subtypes (38, 39). Terashima et al. identified mutations of CCND2 (12p13) and RB1 (13q14), along with the acquisition of PRDM14 (8q13), as factors associated with the development of YST (11). With advancements in next-generation sequencing, frequent abnormalities have been observed in the KIT/Ras and MAPK/AKT/mTOR signaling pathways. Mutations in genes related to the KIT/Ras and PI3K/AKT pathways are present across all intracranial GCT subtypes (40–42). Consequently, future targeted therapies aimed at the MAPK and PI3K pathways may prove effective for refractory intracranial GCTs. Such therapies could be integrated into treatment strategies for certain YST patients to reduce or potentially eliminate the need for radiotherapy. In our study, we observed numerous somatic genomic variations in the patient (**Figures 8C, D**). Notably, genes, such as DDX11 (12p11.21), IGLV2-18 (22q11.22), OR8G5 (11q24.2), PARP4 (13q12.12), USP10 (16q24.1), and ZNF208 (19p12), exhibited multiple mutations and demonstrated tumor-driving effects (**Figure 8B**). For example, Yu et al. found that E2F1 could upregulate DDX11 promoting hepatocellular carcinoma progression via the PI3K/AKT/mTOR pathway (43). Similarly, Xiang et al. showed that the interaction between DDX11-AS1 and HNRNPC enhanced the Wnt/β-catenin and AKT pathways, as well as epithelial–mesenchymal transition (EMT), thereby facilitating glioma cell proliferation and migration (44). Further basic research is essential to fully elucidate the molecular mechanisms underlying YST development.

In terms of treatment, because of its rarity, high malignancy, invasiveness and resistance to radiotherapy and chemotherapy, the prognosis for intracranial YSTs is poor, with a low long-term survival rate. Historical studies show a 1-year survival rate of 33% (34) and a 3-year survival rate of 27% (16). For NGGCT patients who received radiotherapy alone, the overall survival rate was only 20% and 40% (3, 45, 46). Currently, there is still no established standard or criterion for YST treatment. The first-line strategy is complete surgical resection followed by neoadjuvant chemotherapy combined with radiotherapy (16, 47, 48). Common chemotherapy regimens, such as BEP (bleomycin–etoposide–cisplatin) and PBV (cisplatin–bleomycin–vincristine), are platinum based, with four to six cycles recommended. For radiotherapy, the recommended dose of whole-brain total spinal cord radiation (CSI) is 30–36 Gy, with a boost to 54–60 Gy for tumor bed expansion. Secondary surgery is allowed before radiotherapy (49). One month post-surgery, a head CT showed a recurrence of lesions in the right basal ganglia with high density (**Figure 9D**). Due to the patient’s intolerance to chemotherapy and financial constraints, he had only received whole-brain radiotherapy in a local hospital, with over 30 sessions at 40–60 Gy resulting in satisfactory recovery (**Figures 9E, F**). At present, the patient is in stable condition, and we continue to follow up his progress.

In summary, we report a case of intracranial YST initially presenting with basal ganglia hemorrhage. Due to the rarity, slow progression and non-specific clinical course, early MRI signals were unclear, and the absence of a mass led to misdiagnosis. Particularly, the presence of both tumor and hemorrhage can complicate diagnosis raising suspicion of aneurysm or cerebrovascular malformation. In some cases, bilateral basal ganglia lesions may be mistaken for neurodegenerative or autoimmune diseases (50) further delaying optimal tumor treatment. The identification of a slowly progressive lesion in the basal ganglia, along with ipsilateral Wallerian degeneration on MRI and atrophy of the corresponding cerebral hemisphere or brainstem, may serve as early markers of tumor onset warranting further clinical investigation. Future research should aim to elucidate the molecular mechanisms underlying YST progression fostering the development of targeted therapies and promoting a multidisciplinary approach to diagnosis and treatment integrating both chemotherapy and radiotherapy.

## Data Availability

The raw data supporting the conclusions of this article will be made available by the authors, without undue reservation.
